# Clinical, Molecular, and Environmental Risk Factors for Hodgkin Lymphoma

**DOI:** 10.1155/2011/736261

**Published:** 2010-11-29

**Authors:** Alison Maggioncalda, Neha Malik, Pareen Shenoy, Melody Smith, Rajni Sinha, Christopher R. Flowers

**Affiliations:** Winship Cancer Institute, Emory University, Atlanta, GA 30322, USA

## Abstract

Epidemiological studies suggest unique occurrence patterns of Hodgkin lymphoma (HL) worldwide. In most Western countries there is a clear bimodal age distribution with an early peak in young adults followed by a second peak in older adults, particularly among males. In the Middle East and Asia, HL is more common in early childhood. There also are marked racial differences in the presentations of HL and HL subtypes, and particular single nucleotide polymorphisms (SNPs) have been identified as etiological factors suggesting that gene-gene and gene-environment interactions are involved. Personal health choices such as exercise and smoking may modify an individual's chances of developing HL. Numerous studies highlight the impact that exposure to Epstein-Barr virus and other environmental factors have on HL risk. Understanding the relative importance of each of these findings and their links to HL development and survival will help clinical researchers expand curative therapies and create preventative strategies for HL.

## 1. Introduction

Hodgkin lymphoma is a cancer of the lymphatic system commonly characterized by the presence of large, malignant lymphoid cells, Reed-Sternberg cells. HL spreads predictably and contiguously between lymph node groups [1], except for in HIV-positive cases [2] and can be divided into two categories, classic HL (CHL) and nodular lymphocyte predominant (NLP) HL. CHL is further divided into nodular sclerosis (NS), mixed cellularity (MC), lymphocyte-rich (LR), and lymphocyte-depleted (LD) histologic subtypes. In the United States (US), an estimated 8,490 new cases will be diagnosed with HL while HL will be accountable for 1.320 deaths in 2010 [3]. The incidence rates for HL have decreased slightly in men (0.6% per year), but increased slightly in women (0.4% per year) over the past 30 years [3]. According to data from the 2003–2007 Surveillance Epidemiology and End Results (SEER) 17 registries, the incidence rate for HL in US men and women is 2.8 per 100,000 people per year, with white Americans having the highest incidence, followed blacks, Hispanics, and Asian Americans [4]. Although HL can affect children, especially in developing nations, it commonly demonstrates a bimodal distribution with peak incidence among adolescents/young adults and among individuals over 55 years of age [5]. 

As noted above, HL is characterized by the presence of Hodgkin and Reed-Sternberg (HRS) tumor cells, though HRS cells only account for 0.1% to 10% of cells in tissue [6]. HRS cells were determined to be derived from germinal center B cells when researchers found clonally rearrangements in both heavy and light chain immunoglobulin V (IgV) genes, with somatic mutations present [7]. Before this finding, though, the origin of HRS was highly speculative because the cells rarely express typical B-cell markers such as BCR, CD19, CD20, A-myb, and Syk—thus an HRS cell's immunophenotype is not reflective of its origin. In one study of 238 cases (49% NS HL and 27% MC HL), after staining tissue, only 13.8% of patients were CD20-positive. Of those 33 cases, the majority were MC (57.5%) rather than NS HL (36.3%) [8]. Though E12 and E47 (helix-loop-helix transcription factors) are expressed in HRS as expected, the function of these factors are compromised due to the strong expression of their inhibitors, ID2 and ABF1 [9–11]. There is also an absent or low expression of Pax5, the main B-cell lineage commitment factor, in HRS cells [12]. Finally, Notch 1, a transcription factor that promotes T-cell development and inhibits B-cell development of lymphoid precursors, downregulates expression of E2A and EBF, induces ABF1, and binds to Pax5 in HRS tumor cells. All these factors contribute to the lost B-cell phenotype in HRS cells. 

Treatment strategies for HL stratify patients into categories based upon prognostic factors, which include stage (as classified by the Ann Arbor staging system or its Cotswold modification), age, anemia, and sedimentation rate [13]. The modern standard combination chemotherapy regimen for HL is adriamycin, bleomycin, vinblastine, dacarbazine (ABVD), which has usurped previous treatment strategies such as radiation therapy alone and nitrogen mustard, vincristine, procarbazine, prednisone (MOPP). Risk-adapted therapy also may be utilized in which patients who have a worse prognosis are given more aggressive treatment at the outset [13]. In the 1960s, the 5-year survival rate for HL was less than 10% [14]. Based on SEER 2003–2007 data, the 5-year relative survival rates for white men and women were 84% and 86%, respectively, while the rates for black men and women were 78% and 86%, respectively [4]. Thus, both HL incidence and survival rates are associated with gender, race, and age. Despite the emerging data on the origins of HRS cells, there are no preventative measures or early interventions for HL. Only by studying this disease's epidemiology and etiology can we further improve therapies, methods for early detection, and prevention. In this paper, we review the studies on epidemiology and the risk factors for HL.

## 2. Patterns of HL Incidence across Populations

International population-based studies highlight regional, racial, ethnic, and socioeconomic differences in the incidence of HL around the world. Associations between clinical symptoms, features, and incidence of HL in a given population could provide genetic and environmental insight into disease etiology. Population-specific analyses of HL incidence in Singapore, Israel, Canada, the United States Asian population, and a multiethnic region of the United Kingdom offer insights into racial, ethnic, and socioeconomic variability in the incidence and patterns of presentation for HL and HL subtypes.

### 2.1. Singapore

As noted above, HL has been characterized by a bimodal, age-specific, incidence pattern with a high proportion of cases occurring in adolescents and young adults, in Western industrialized countries [15]. In order to analyze whether the young adult peak is associated with an affluent socioeconomic environment occurring as a byproduct of growing up in industrialized countries, Hjalgrim et al. evaluated HL incidence pattern in Singapore between 1968 and 2004, a period when Singapore experienced a major socioeconomic shift towards Western lifestyle [16]. Using the Singapore Cancer Registry, researchers identified a cohort of 193 women and 337 men diagnosed with HL during the study period. Among men, HL incidence rates increased annually from 1968 to 2004 by 7% (95% Confidence Interval [CI] 3.4%–10.7%) and by 3.4% (95% CI 0.1%–6.8%) for the 15 to 19 year-old and the 20 to 24 year-old age groups, respectively. The trend was even more pronounced among women in these age groups with increases of 13.7% (95% CI 9.1%–18.6%) and 12.2% (95% CI 7.8%–10.8%), respectively. Overall, however, the incidence peak for young adults remained lower than that of young adults in the Western world. Researchers speculate that birth cohort phenomenon and ethnic genetic variation between Asians and non-Asians may contribute to these differences. 

### 2.2. Israel

Using data from the Israel Cancer Registry on 4,812 HL cases diagnosed between 1960 and 2005, Ariad et al. found that the reported incidence level of HL in Israeli-born young adult Jews increased in recent years and surpassed that of any other western country [27]. To build on a previous study that found a high incidence of Epstein-Barr virus (EBV) expression in HL tumor cells in the Bedouin people, Levy et al. examined HL cases in 4 community subgroups in Southern Israel: Kibbutz residents (*n* = 11), Bedouin (*n* = 19), new immigrants from the former USSR (*n* = 22), and town-dwellers (*n* = 82) [28]. The study explored clinical features, demographic data, stage at diagnosis, treatment modality and outcome, and laboratory findings, and EBV expression in each of the groups. Bedouin patients were significantly younger than the rest of the cohort when diagnosed with HL, and the Bedouin group had a higher proportion of males than any other group (79% versus 53%). Additionally, a greater proportion of Bedouin patients presented with the MC HL subtype (*P* = .16), presented with advanced disease (85.7%, *P* = .04), and were resistant to treatment (47.4% versus 27.7%, *P* = .09) compared to the other groups. EBV expression was significantly more frequent, and mdm-2 (murine double minute oncogene, a negative regulator of the p53 tumor suppressor) was less frequent in the Bedouin than in any other group [29]. However, interpreting the significance of these differences is difficult given the small sample size of the study.

### 2.3. Canada

Pahwa et al. investigated the association between ethnicity and incidence of HL in the Canadian male population, taking into account medical history and exposure to pesticides, to better understand both genetic and environmental etiological factors [17]. Using a postal questionnaire and telephone interview, they gathered information from 316 Canadian men, over the age of 19, diagnosed with HL between 1991 and 1994 and a random sample of 1506 control subjects matched by age (±2 years) and gender. The authors defined ethnicity as having 3 out of 4 grandparents born in the same ethnic region, defined pesticide exposure based on a pilot study, and acquired a medical history. When compared with people of North American descent, people of Eastern European descent had a greater risk of HL (Odds Ratio adjusted [ORadj] 1.82, 95% CI 1.02–3.25, Table 1), while Asian descendants had a significantly lower risk (ORadj 0.17, 95% CI 0.02–1.33). History of measles (ORadj 0.72, 95% CI 0.53–0.98) and allergy desensitization shots (ORadj 0.55, 95% CI 0.30–0.99) were negatively associated with HL incidence, whereas acne (ORadj 2.12, 95% CI 1.19–3.78), shingles (ORadj 2.41, 95% CI 1.38–4.22), and a family history of cancer (ORadj 1.93, 95% CI 1.40–2.65) were positively associated with HL incidence in this cohort. Additionally, exposure to the herbicide dichlorprop showed an increased risk of HL (ORadj 6.35, 95% CI 1.56–25.92).

### 2.4. United States: Asian Population

Based on reports that Asians have consistently low rates of HL incidence, Glaser and Hsu explored individual genetic and environmental risk factors in both US-born and foreign-born Asian populations (Chinese, Japanese, Filipino, and Asian Indian) [30]. Glaser and Hsu analyzed 225 US-born cases and 1,477 foreign-born cases with data from the US SEER cancer registry, California Cancer Registry, and the International Agency on Cancer registry, all of which had preassigned ethnicity according to their own standards. HL incidence rates were relatively low for all Asians when compared to whites, but US-born Asians had approximately double the incidence when compared to native Asians. For US-born Chinese, Japanese, Filipino, and Asian Indian men, the annual age-adjusted incidence rates per 100,000 individuals in the population were 0.7, 0.8, 1.4, and 2.9, respectively. For native Asians, the incidence rates were 0.5, 0.5, 0.9, and 1.3, respectively. US-born Asian women also had higher incidence rates than native Asian women (0.4 versus 0.3 for Chinese, 0.6 versus 0.2 for Japanese, 1.0 versus 0.4 for Filipino, and 1.5 versus 0.6 for Asian Indians, resp.). Additionally, a young adult incidence peak occurred only in the US-born Asian population. According to Glaser and Hsu, consistently low incidence rates of HL in the Asian population as a whole suggest genetic resistance, possibly related to HLA type, and differences between US and native Asian groups suggest environmental influences. 

### 2.5. United Kingdom

Flavell et al. analyzed the effects of ethnicity and material deprivation on 55 pediatric patients (under 15 years of age) with HL as well as the influence of EBV on HL incidence [31]. Researchers acquired data from the West Midlands Regional Children's Tumor Registry in a multiethnic region of the United Kingdom. The patients were separated into two ethnic groups based on self-reported ethnicity: South Asians (of Indian, Pakistani, or Bangladeshi descent) and non-South Asians (white, black, Hispanic, Oriental, or of mixed race). Sixty-two percent of all HL cases were EBV-positive in this study population. EBV-positivity was strongly associated with ethnic group, with 18 of 19 (95%) South Asian cases being EBV-positive, while only 16 of 36 (44%) non-South Asian cases were EBV positive (*P* < .001). Based on these data South Asians had more than a 20-fold increased risk of EBV-positive HL when compared to non-South Asians. 

This group also investigated the impact of material deprivation on HL as determined by postal zip code and measured with the associated Townsend score. Townsend score was determined by the percent of unemployed residents, percent of households without cars, percent of households not owner-occupied, and percent of households with more than one person per room yielding a possible range of −12 to +12 (by combining *z*-scores from −3 to +3 for each of the four factors) [32]. EBV-positivity was associated with higher deprivation scores (median Townsend score +3.2), whereas EBV-negativity was associated with lower scores (median score −0.4). As material deprivation increased (among Townsend score quartiles), so did the proportion of EBV-positive HL cases (*P* = .025). The risk for EBV-positivity was 7 times higher in the most deprived quartile than the least. After adjusting for ethnicity, risk was still 3 times higher. After adjusting for Townsend score, ethnicity was the only factor independently associated with EBV-positivity (OR 10.9, 95% CI 1.20–98.6). These data suggest that ethnicity and material deprivation independently influence the development of EBV-associated HL.

## 3. Risk Factors for HL

### 3.1. Socioeconomic Status

The population-based studies by Hjalgrim et al. and Flavell et al. discussed above further elucidated the role that socioeconomic status (SES) plays in HL incidence [16, 31]. As noted above, the shift towards Western lifestyle in Singapore was accompanied by a shift in HL incidence, and in the UK, patients who had the highest rates of material deprivation (lowest SES) had a three-time higher risk for EBV-positive HL. Additional studies have investigated the effects of socioeconomic factors such as education, income, and ownership of goods on HL incidence and survival. In Denmark, Roswall et al. studied the association between social inequality and incidence of HL and NHL, and leukemia in 8,638 cases in comparison to a cohort of 3.22 million people, the Denmark population [33]. The investigators used data from individuals born between 1925 and 1973 and diagnosed with HL between 1994 and 2003. Socioeconomic status was determined by means of a questionnaire inquiring about education level, disposable income, social class, housing tenure, and the size of dwelling. Comorbidity was measured with the Charlson comorbidity index. There was no clear evidence of a relationship between disease incidence and SES, but there was an association between comorbidity and overall survival for all three diseases. Among HL patients (*n* = 636), age-standardized relative survival was worst for women with basic education and men with vocational education. A Brazilian study also found that higher SES was associated with a better 2-year survival rate than lower SES (93% versus 79%, *P* = .01) [34].

### 3.2. Occupational Exposures

Karunanayake et al. investigated the association between occupational exposures and risk of HL among 18-to 92-year old Canadian males in order to better understand the potential epidemiological underpinnings of HL [19]. Using postal questionnaires and telephone interviews, researchers acquired occupational and medical data from 316 cases of HL and 1506 controls. Exposure was defined as ever having contact with dust, coal products, printing products, paints, metals, radiation (divided into nonionizing and ionizing), and other products. Long-held occupation was defined as having worked for 10 or more years in given occupation. Though there was no significant difference between HL risk and long-held occupation (whether accountant, clerk, driver, farmer, engineer, manager, or salesman), exposure to ionizing radiation, history of certain medical illness, and habit of ever smoking were associated with risk of HL. Exposure to ionizing radiation from uranium significantly increased risk of HL (OR 2.58, 95% CI 1.08–6.19). Exposure to ultraviolet light (OR 0.62, 95% CI 0.4–0.96) and diagnosis of measles (OR 0.7, 95% CI 0.53–0.94) was negatively associated with HL, whereas a diagnosis of shingles was positively associated with HL (OR 2.16, 95% CI 1.31–3.56). Subjects with any history of smoking for 25 or more years were 1.9 times likely to be diagnosed with HL than nonsmokers. These data are corroborated by studies on exposure to pathogens and environmental tobacco smoke described below.

### 3.3. Childhood Environment

An individual's childhood social environment may influence bacterial exposure which, in turn, can provide clues as to the pathological etiology of HL. Chang et al. investigated the association between childhood social environment, namely, exposure to childhood pathogens and HL in order to better understand the disease's viral etiology [18]. Researchers surveyed 565 HIV-negative HL cases and 679 controls in Massachusetts and Connecticut and inquired about level of parental education, homeownership status, number of siblings, housing density, birth order, and nursery school/day care attendance. All questions, with the exception of nursery school attendance, were answered with the reference age of 8 years old. For 15 to 54 year olds (first incidence peak), HL cases were more likely than controls to be Jewish rather than Catholic (OR 2.29, 95% CI 1.26–4.19) or Protestant (OR 1.77, 95% CI 1.01–3.13), to have smoked ≥10 lifetime packs of cigarettes (OR 1.31, 95% CI 1.03–1.69), to be less educated (OR 0.82, 95% CI 0.70–0.96), and to have a family history of hematopoietic cancer (OR 2.06, 95% CI 1.10–3.87). In this same age group, attendance of nursery school or day care lowered HL risk by 13%. If individuals attended day care for more than 1 year, the risk of HL dropped by 36%. These associations were not consistent for the older age group. Among 55 to 79 year olds, HL risk was associated with lower childhood socioeconomic status. Parental education, number of siblings, history of infectious mononucleosis, and housing density had no significant correlation with HL despite previous findings in other studies.

In another study, young adult Hodgkin lymphoma (YAHL) has been linked to lower levels of interleukin 12 levels and persistence of the T-helper type 2 immature immune response phenotype [35, 36]. Because YAHL is associated with a Th-2 skewed immune response [37, 38], Cozen et al. investigated the relationship between early microbiological exposures that affect Th-2/Th-1 cytokines and the incidence of YAHL [20]. Researchers sent 35-page questionnaires to 188 sets of twins, one with YAHL and one without (found through a North American twin study [39]) and asked about early infections, surgical, medical, and pharmacological history, alcohol and tobacco use, oral exposure habits such as sucking one's thumb, education, and puberty. The reference date for these questions was five years before the diagnosis of YAHL as to avoid answers associated with disease development. Prior appendectomy (*P* = .001), eczema (*P* = .025), and smoking (*P* = .054) were associated positively with YAHL incidence, whereas childhood behaviors associated with oral exposure (thumb-sucking, toys in mouth, etc.) were inversely associated with the disease (*P* = .004). These investigators concluded that early exposure to microbiomes may protect against onset of YAHL because exposure affects cytokine balance. 

### 3.4. Personal Health Choices: Exercise, Medication and Alcohol Use, and Smoking Exercise

Social environment and peer influence affect a variety of personal health choices, from exercise and taking certain medication to smoking cigarettes and drinking alcohol, that are also associated with HL risk. Keegan et al. studied height, weight, body mass index, and strenuous physical activity in association with the risk of HL in women in 312 cases and 325 controls aged 19 to 79 years, who self-reported these measurements as well as reproductive factors, exposure to exogenous hormones, and SES [22]. Strenuous physical activity was defined as “getting out of breath or working up a sweat” at least twice a week. In young women, 19 to 44 years of age, participation in strenuous physical activity for at least one month before reference period resulted in approximately a 40% reduction in odds of HL. The odds of developing HL were significantly lower with any participation in strenuous physical activity over the past year in young women (OR 0.58, 95% CI 0.39–0.87) but not older women, 45 to 79 (OR 0.45, 95% CI 0.19–1.06). The relationship between being overweight, versus normal weight, with respect to HL risk was significantly different between older and younger women (*P* = .02). Young women in higher body mass index (BMI) quartiles had increased risk (ORadj 1.74, 95% CI 1.00–3.02 for 4th versus 1st quartile), whereas older women with higher BMIs were associated with lower risk (ORadj 0.37, 95% CI 0.11–1.30 for 4th versus 1st quartile). Additionally, taller height was significantly associated with higher risk of HL (*P*  for trend = .01). Gunnell and Okasha suggest that higher levels of insulin-like growth factors and/or other growth hormones found in taller women could potentially be related to HL tumor growth [40].

### 3.5. Aspirin Intake

Chang et al. analyzed recent findings of an inverse association between routine use of a regular strength aspirin and HL risk and explored investigations of selective cyclooxygenase-2 inhibitors and nonaspirin nonsteroidal anti-inflammatory drugs (NSAIDs) in order to determine whether or not aspirin truly does have a protective property against HL [23]. With the aid of Denmark's nationwide cancer registries and prescription databases, researchers identified 478 cases and 10 controls for each case with fully disclosed medication histories. Prescription users were defined as subjects with two or more prescriptions of aspirin and/or other NSAIDS. Chang et al. found that low-dose aspirin use was indeed inversely associated with HL risk but only among never/rare users of nonaspirin NSAIDs (OR 0.6, 95% CI 0.3–1.0). There was no significant association between selective cyclooxygenase-2 inhibitors or other NSAIDs and HL risk. 

After finding that regular use of low-dose aspirin may reduce risk of HL, Chang et al. performed another study to examine the biological underpinnings of this phenomenon [25]. The study investigated polymorphic variation in genes involved in nuclear factor-*κ*B (NF*κ*B) activation/inhibition, other inflammatory pathways, and aspirin metabolism. Researchers analyzed 20 SNPs in 7 genes of 473 HL cases and 373 controls. To determine target genes, the team used a candidate-SNP approach and utilized both literature and database information. HL risk was significantly associated with rs1585215 polymorphisms in NFKB1 (AG versus AA: OR 2.1, 95% CI 1.5–2.9; GG versus AA: OR 3.5, 95% CI 2.2–5.7, Ptrend = 1.7 × 10^−8^) and NFKB1 haplotypes (Pglobal = 6.0 × 10^−21^). Marginally weaker associations were found between HL risk and SNPs with NFKB1A and CYP2C9, whereas no association was found with IKKA/CHUK, PTGS2/COX2, UDP1A6, or LTC4S. Based on the data, Chang et al. suggests NFKB genetic involvement in HL pathogenesis.

### 3.6. Smoking and Environmental Tobacco Smoke

In a French case-control study, Monnereau et al. investigated the relationship between cigarette smoking, alcohol consumption, and risk of lymphoid neoplasm among patients with HL, non-Hodgkin lymphoma (NHL), multiple myeloma (MM), and lymphoproliferative syndrome [41]. The research team sent questionnaires to 824 cases and 752 controls, aged 18 to 75 years, and asked about smoking status, tobacco and cigarette type, smoking duration, number of cigarettes per day, lifelong alcohol consumption, and number of drinks per week. A subject was considered a smoker if he or she had ever had at least one cigarette per day for more than 6 months, a current smoker if he/she was doing so at the time of interview. A subject was considered a drinker if he/she routinely had one drink (beer, wine, aperitif, etc.) per month. Overall, smoking was not associated with increased risk of lymphoid neoplasms (OR 0.6, 95% CI 0.4–0.9). However, average tobacco consumption was inversely related to lymphoproliferative syndrome and NHL (Ptrend < .05). This inverse relationship was especially prevalent between smoking and hairy cell leukemia (HCL) (OR 0.2, 95% CI 0.1–0.5). There was also an inverse association between drinking alcohol and HL (OR 0.5, 95% CI 0.3–0.8). These data could be interpreted to suggest a protective effect of smoking and alcohol consumption with respect to HL and NHL; however, fairly broad definitions for both exposures were used.

Although this study did address a potential association between smoking and lymphoma incidence, in separate studies Briggs et al. and Glaser et al. studied the relationship between cigarette smoking and HL in men and women, respectively, in much more depth [21, 24]. They examined factors such as smoking age onset, duration, intensity, and even environmental tobacco smoke exposure. To address the lack of information on the relationship between smoking and HL in men, Briggs and colleagues investigated whether a dose-response effect of smoking in relation to risk of HL exists in the hope of discovering a potential preventable epidemiological factor for the disease [24]. Using data from the Selected Cancers Study, researchers conducted 2,104 telephone interviews with 308 American men diagnosed with HL and 1,796 controls, all between the ages of 32 and 60. Researchers considered smokers as subjects who answered “yes” to the following question: “Did you ever smoke at least a 0.5 pack of cigarettes weekly for at least a year?” Smokers were asked about intensity (packs/day), duration (years), onset age, and years since quitting if applicable. Compared to never-smokers, current smokers had a significantly increased risk of HL (OR 1.8, 95% CI 1.3–2.9). Furthermore, risk increased linearly with increase of packs/day (OR 2.5, 95% CI 1.6–4.0), years (OR 2.4, 95% CI 1.5–3.9), and pack-years (OR 2.7, 95% CI 1.8–4.3) of smoking. Similar associations were observed for nodular sclerosis HL and were even more profound for mixed cellularity HL. Additionally, former smokers, when compared to current smokers, had a significant and linear decrease in risk of HL with increasing time since having quit. This was true for risk of HL overall and for HL subtypes. 

With the belief that smoking had been considered a risk factor for HL only among men, Glaser et al. performed a population-based, case-control study, analyzing the effect of smoking and household environmental tobacco smoke (ETS) exposure on women (aged 19 to 79) in the Greater San Francisco Bay Area [21]. Researchers interviewed 95% of the 637 subjects (312 diagnosed with HL and 325 randomly selected controls) in person and 5% by telephone, asking standardized questions about frequency, duration, and intensity of smoking, if any, and exposure to ETS, age on onset, and duration of ETS. Because EBV is believed to play a role in HL etiology, researchers also tested all possible subjects for tumor-cell EBV and incorporated those findings into their results. Among 253 cases compared to 254 control, aged 19 to 44 years, exposure to ETS in childhood was associated with a 50% increased risk for HL overall (OR 1.7, 95% CI 0.9–3.4). In 24 cases of EBV-positive HL, current smoking, greater smoking intensity (*P* = .05 for current smokers, *P* = .02 for ever-smokers) and duration (*P* = .04 for current smokers, *P* = .06 for ever-smokers), and ETS exposure also increased risk. Most smoking characteristics did not seem to affect HL risk in the other 59 cases and 71 controls, aged 45–79.

## 4. Impact of Epstein-Barr Virus and Human Immunodeficiency Virus on HL Incidence

### 4.1. Epstein-Barr Virus

Because viral infection can damage DNA and alter cell composition in healthy cells, investigators have examined the relationships between viral infection, T-cell function, and the development of HL. For a case to be determined as EBV-positive HL, EBV nucleic acids and proteins must be detectable in tumor cells [42]. Although in North America and Western Europe, 30%–50% of HL cases are EBV-positive, EBV is detected in a relatively small proportion of RS/H tumor cells. In parts of Latin America, Africa, and Asia, however, EBV-positivity in children with HL approaches 100% [43]. Research led by Dr. Ambinder suggests that this phenomenon is due to primary infection of EBV and infectious mononucleosis being a potential precursor to EBV-associated HL [42]. 

Although most individuals are EBV seropositive with antibody responses reflecting a prior exposure to EBV, most individuals have subclinical exposures. In the United Kingdom, one study followed 500 seronegative college students. After 3 years, 46% of those students converted to being seropositive. Of those students, 25% developed infectious mononucleosis [44]. Infectious mononucleosis was first linked with HL in the 1950s, but in the 1980s viral antigens were found in HL RS/H cells [42]. Oddly enough, association between infectious mononucleosis and HL was most prevalent in young adults, although virus presence in tumor cells was least frequent for this age group [42]. In a Scandinavian study, researchers found an association between HL and infectious mononucleosis with HL tending to occur 2.9 years after occurrence of infectious mononucleosis [45].

Additional studies have aided in our understanding of the molecular biology of virus-associated HL [46]. EBV-positive HRS cells express EBNA1, key for viral genome replication, as well as LMP1 and LMP2a which mimic CD40 receptors and BCRs, respectively [47, 48]. Additionally, virtually all HL cases with BCR preventing destructive IgV mutations are EBV-positive [49]. The Epstein-Barr virus rescues BCR-mutated GC B cells from apoptosis with LMP2a expression which leads to EBV-positive HRS cells [48, 50, 51]. In essence, this finding suggests that the virus facilitates cancer cell proliferation. Finally, TNFAIP3 is a tumor suppression gene that encodes for A20, a tumor suppressing protein. In EBV-negative HL cases, 70% of patients had destructive TNFAIP3 mutations. In EBV-positive cases, however, only 12% of patients had TNFAIP3 mutations, and the majority were missense mutations [52]. These findings suggest that EBV has a distinct pathogenic effect on EBV-positive HL [46]. Crippled immunoglobin genes responsible for the prevention of apoptosis of cancer cells are not often found in HL but, when they are, they are almost always found in EBV-positive HL cases. 

In an effort to study EBV-positive HL incidence on an international scale, Glaser et al. analyzed data from the US, UK, France, Denmark, Germany, Argentina, and China [43]. Researchers compared EBV-related HL prevalence in relation to age of diagnosis, sex, ethnicity, histologic subtype, country of residence, and clinical stage in 1,546 patients. EBV prevalence across four age groups (0–14, 15–39, 40–54, and 55+) was higher among Asians (92.9%, 38.1%, 50.0%, 81.8%) and Hispanics (85.7%, 49.3%, 63.2%, 66.7%) than among whites (46.2%, 29.6%, 37.6%, 48.3%) and blacks (16.7%, 13.0%, 33.3%, 20.0%). Almost twice as many males had EBV-related HL than women (47.7% versus 29.2%). The highest proportion of EBV-positive HL cases was in the oldest and youngest age groups. In terms of histologic subtype, MC had the largest proportion of EBV-positive HL, and LP had the smallest (70.4% and 16.0%, resp.). Additionally, patients from less developed countries were twice as likely to be EBV-positive than those from more developed countries (63.4% versus 36.0%). EBV-related HL was most prevalent in Saudi Arabia (87.5%) and least prevalent in the United Kingdom (30.8%).

Rather than studying EBV influence on incidence, Keegan et al. assessed the joint effects of EBV with age, sex, and histologic subtype on survival after HL diagnosis [53]. The study included 922 HL cases among children and adults in the Greater San Francisco Bay Area who were diagnosed between 1988 and 1997. For children under 15 years of age, EBV-positivity was associated with favorable survival (*P* = .07; HR (hazard ratio) 0.18, 95% CI = 0.02–1.70). EBV status did not affect outcome for cases between the ages of 15 and 44, though data suggested a potential protective effect. For 45- to 96-year old patients with the nodular sclerosis subtype of HL, however, EBV presence nearly doubled risk of death (*P* < .01; HR 2.50, 95% CI 1.50–4.30). Data suggested that EBV presence played a significantly different role in survival between children and older adults with HL.

### 4.2. Human Immunodeficiency Virus

Another form of virus-related HL is human immunodeficiency virus- (HIV-) positive HL. Characteristically, HIV-HL patients present at a more advanced stage with associated extranodal involvement and B-symptoms. Additionally, the majority of HIV-HL cases are of the MC HL subtype, whereas NS HL is most common for HIV-negative patients [2]. Another distinct feature of HIV-positive HL is the noncontiguous spread of disease; some patients even have bone-marrow only presentation. Biggar et al. found that people with HIV/AIDS were also more likely than the general population to be diagnosed with HL. AIDS cases with moderate immunosuppression (225–249 CD4 cells/*μ*L at onset) were estimated to have a 15-fold increased risk of HL [54].

Glaser et al. examined the effect of risk factors: EBV association, incidence rates, and survival rates among HIV-related HL in San Franciscan men [2]. Researchers analyzed data on 1,752 patients with HL; 128 of which were HIV-positive, from the Greater Bay Area Cancer Registry from 1988–1998. Because 95% of HIV-related HL cases were men, all conclusions are based on data regarding men only. HIV-positive patients were more likely than HIV-negative patients to exhibit extranodal disease (67% versus 32%), B-symptoms (77% versus 43%), and advanced Ann Arbor stage III or IV disease (82% versus 42%). In addition, HIV-positive and HIV-negative HL estimated survival rate probabilities after 1 year, 2 years, and 5 years of diagnosis were 69% versus 92%, 56% versus 87%, and 38% versus 78%, respectively. HIV-positive HL patients were more likely to be black than white (*P* = .02) and more likely to have MC HL, LD HL, or CHL NOS subtypes than have NS HL. The 38 HIV-positive HL patients with NS HL had a significantly better chance of survival than those with other subtypes (*P* = .03). Among patients with the HL NOS subtype, risk of HIV-related disease was higher in blacks than whites (OR 4.40, 95% CI 0.90–21.5). Additionally, EBV was detected in 90% of the 59 HIV-positive patients, whereas only 32% of the 473 HIV-negative patients were EBV-positive. HIV-positive HL cases diagnosed after 1996, when highly active antiretroviral therapies (HAART) came into play, had a marginally better survival rate than those diagnosed before the HAART era (*P* = .07).

### 4.3. Molecular Biology of Virus-Related HL

Mollaki et al. studied the risk of Hodgkin lymphoma in relation to TLR9-1237T>C, TLR9 2848A>G, MYD88-938C>A, and MYD88 1944C>G gene polymorphisms and haplotypes [26]. These genetic variants play roles in virus recognition and prevention of viral cell proliferation and thus may be related to viral-mediated diseases. Using tissue samples from three hospitals in Greece, researchers examined single-nucleotide polymorphisms (SNPs) from 90 tissue samples of patients with HL and 92 healthy controls. Polymerase chain reaction (PCR) was used to amplify DNA fragments of interest. Researchers determined that carriership for −1237C and 2848A was associated with increased HL risk (OR 2.53, 95% CI 1.36–4.71 and OR 6.20, 95% CI 1.30–28.8, resp.). Between HL cases and controls, estimated frequencies of TLR9/1237C-2848A and MYD88/938C-1944G haplotypes were significantly different (*P* < .01), as were observed TLR9/1237C-TLR9/2848A-MYD88/938C-MYD88/1944C haplotype differences between the two groups (*P* < .01). These findings suggest that TLR9 and MYD88 haplotypes and TLR polymorphisms are associated with HL development.

Chetaille et al., members of the French network Groupe d'Etude des Lymphomas de I'Adulte, aimed to characterize the microenvironment of CHL [55]. Researchers used 63 CHL tissue samples (10 from children) and profiled them using DNA microarrays. Histiocyte T-cell-rich B-cell lymphoma tissue was used as a control because H/TCRBCL and CHL have similar amounts of reactive cells and macrophages in the stroma. Unlike CHL, H/TCRBCL tissues exhibited high expression of PCD1/PD-1, a gene that codes for lymphocyte inhibitory receptors. Samples also showed that the presence of EBV was readily distinguishable with gene signature characteristic of Th1 and antiviral responses (GS9 and GS11, *P* < .001; G10, *P* = .003). Genes overexpressed in EBV-positive CHL include those involved in innate immune response (*P* = .018), immune response (*P* = .005), and defense response (*P* = .007).

Hjalgrim et al. investigated the association between HLA-A*01 and HLA-A*02 in the setting of infectious mononucleosis (IM) and EBV-related HL to determine whether or not HLA class-I restricted EBV-specific cytotoxic T-cell responses and early immune response to EBV infection in IM affect pathogenesis of EBV-related HL [56]. Researchers analyzed 278 EBV-related HL cases and 656 EBV-unrelated HL controls in a case-series analysis. When comparing genotypes of the two groups to confirm that EBV-unrelated HL patients would be appropriate controls, the difference in HLA-A*01 homogeneity was significant, reflecting a small number of heterozygotes among EBV-unrelated HL. 

Among individuals never diagnosed with IM, there was nearly tenfold odds variation between HLA-A*01 and HLA-A*02 homozygotes in risk of EBV-related HL (OR 9.45, 95% CI 4.6–19.4). Overall, HLA-A*01 alleles were associated with increased risk of EBV-related HL (OR 2.50, 95% CI 1.60–2.88), whereas HLA-A*02 alleles were associated with decreased risk (OR 0.70, 95% CI 0.51–0.97). Researchers also found a positive association between history of IM and EBV-related HL, though only in patients who lacked both HLA-A alleles (OR 2.82, 95% CI 1.15–6.90). Additionally, history of IM was independently associated with risk of EBV-related HL, though not in the presence of HLA-A*02 which appeared to neutralize effect of IM on risk (OR 3.40, 95% CI 1.74–1.66).

## 5. Conclusions

Epidemiology studies suggest a unique occurrence pattern of HL. In most Western countries there is a clear bimodal age incidence with an early peak in young adults followed by a second peak in older adults, particularly among men. Similar trends were recently demonstrated in a SEER study [57]. From our review of the literature, it appears that in the Middle East and parts of Asia the peak is more pronounced in early childhood. There are also clear clinical and laboratory differences across smaller communities in certain areas as seen in the study conducted in Southern Israel [28].

Our paper suggests that SES is both a potential risk factor and survival predictor for HL. A socioeconomic shift towards Western lifestyle was accompanied by an increased HL incidence in Singapore [15]. Lower SES, on the other hand, was associated with a worse prognosis. Several studies conducted in the western world also highlight the impact of environmental factors that can increase HL risk [33, 34]. Childhood exposure to pathogens, microbes, and microbiomes can potentially reduce the risk of HL [18, 20]. Childhood day care attendance and innate behaviors such as thumb-sucking and putting toys in the mouth have been inversely associated with HL risk which further suggests that early pathogen exposure might be beneficial to healthy development in children [35, 36]. Additionally, there appear to be marked racial differences of HL and HL subtypes, and particular SNPs have been identified as etiological factors suggesting that gene-gene and gene-environment interactions are involved.

Personal health choices can be made that may decrease individual's chances of developing HL. Studies have found that strenuous exercise at least twice a week can decrease the risk of HL by approximately 40% [22]. Educating children, adolescents, and adults about the benefits of routine exercise along with the risks associated with lack of exercise may help to reduce the incidence of HL in those age groups. Passive and active smoking is another lifestyle choice that has been associated with a higher risk of HL [24]. One study found that just being exposed to cigarette smoke as a child increased HL risk by approximately 50% [21]. The important findings of these possible links to HL allow individuals to opt for lifestyle modifications that may reduce the risk of developing HL and other diseases. 

It has been reported that HL may be a rare consequence of a common infection, with the probability of oncogenesis increasing with age at the time of infection. Patients with EBV exposure, which may clinically present itself as IM, are at a higher risk of HL due to the molecular biology of the virus [42, 48, 50, 51]. However, relatively few individuals exposed to EBV or with IM ultimately develop HL. It has also been documented that HIV-positive HL patients have more advanced disease at presentation and poorer survival than those with HIV-negative [2]. The molecular biology of virus-related HL is still not well understood but attempts at isolating specific transcription factors and genes associated with the disease are underway. Additional research on the oncogenesis of HL and mitigating genetic and environmental factors may help clinical researchers expand curative therapies and create preventative strategies for HL.

##  Disclosures

None of the authors have conflict of interests.

## Figures and Tables

**Table 1:** Risk factors for Hodgkin lymphoma.

Risk Factor	Odds Ratio	95% CI	Study
*Race/Ethnicity*			
E European versus N American descent	1.82	1.02–3.25	Pahwa [17]
Jewish versus Catholic	2.29	1.26–4.19	Chang [18]
Jewish versus Protestant	1.77	1.01–3.13	Chang [18]

*Medical History*			
Measles	0.72	0.53–0.98	Pahwa [17]
	0.7	0.53–0.94	Karunanayake [19]
Shingles	2.41	1.38–4.22	Pahwa [17]
	2.16	1.31–3.56	Karunanayake [19]
Appendectomy	4.3	1.90–10.00	Cozen [20]
Acne	2.12	1.19–3.78	Pahwa [17]
Allergy desensitization shots	0.55	0.30–0.99	Pahwa [17]
Eczema	4.2	1.20–14.80	Cozen [20]
Family history of cancer (general)	1.93	1.40–2.65	Pahwa [17]
Family history of cancer (hematopoietic)	2.06	1.10–3.87	Chang [18]

*Exposure History*			
Herbicide dichlorprop	6.35	1.56–25.92	Pahwa [17]
Ionizing radiation from Uranium	2.58	1.08–6.19	Karunanayake [19]
Ultraviolet radiation	0.62	0.40–0.96	Karunanayake [19]
Childhood environmental tobacco smoke (F 19–44 years)	1.7	0.90–3.40	Glaser [21]
Behaviors (thumb-sucking/putting things in mouth) resulting in early oral exposure than twin	0.1	0.00–0.50	Cozen [20]
≥1 year nursery school attendance	0.64	0.45–0.92	Chang [18]

*Physical Health*			
2X/wk physical activity (F 19–44 years)	0.58	0.39–0.87	Keegan [22]
2X/wk physical activity (F 45–79 years)	0.45	0.19–1.06	Keegan [22]
High BMI (F 19–44 years)	1.74	1.00–3.02	Keegan [22]
High BMI (F 45–79 years)	0.37	0.11–1.30	Keegan [22]
Low-dose aspirin use	0.6	0.30–1.00	Chang [23]
Smoking ≥10 lifetime packs of cigarettes	1.31	1.03–1.69	Chang [18]
Current smoker (M)	1.8	1.30–2.90	Briggs [24]

*Molecular Biology*			
rs1585215 in NFKB1: AG versus AA	2.1	1.50–2.90	Chang [25]
rs1585215 in NFKB1: GG versus AA	3.5	2.20–5.70	Chang [25]
Carriership for −1237C (TLR9)	2.53	1.36–4.71	Mollaki [26]
Carriership for 2848A (TLR9)	6.20	1.30–28.8	Mollaki [26]

*Socioeconomics*			
Having less than a high school education (15–54 years)	0.82	0.70–0.96	Chang [18]

E: East; N: North; F: female; M: male, BMI: body mass index.
